# Molecular and Cellular Features of Murine Craniofacial and Trunk Neural Crest Cells as Stem Cell-Like Cells

**DOI:** 10.1371/journal.pone.0084072

**Published:** 2014-01-20

**Authors:** Kunie Hagiwara, Takeshi Obayashi, Nobuyuki Sakayori, Emiko Yamanishi, Ryuhei Hayashi, Noriko Osumi, Toru Nakazawa, Kohji Nishida

**Affiliations:** 1 Department of Ophthalmology, Tohoku University Graduate School of Medicine, Seiryo-cho, Aoba-ku, Sendai, Japan; 2 Division of Applied Informatics for Human and Life Science, Tohoku University Graduate School of Information Science, Aramaki-Aza-Aoba, Aoba-ku, Sendai, Japan; 3 Division of Developmental Neuroscience, Tohoku University Graduate School of Medicine, Seiryo-cho, Aoba-ku, Sendai, Japan; 4 Department of Ophthalmology, Osaka University Graduate School of Medicine, Yamadaoka, Suita, Japan; Tokyo Medical and Dental University, Japan

## Abstract

The outstanding differentiation capacities and easier access from adult tissues, cells derived from neural crest cells (NCCs) have fascinated scientists in developmental biology and regenerative medicine. Differentiation potentials of NCCs are known to depend on their originating regions. Here, we report differential molecular features between craniofacial (cNCCs) and trunk (tNCCs) NCCs by analyzing transcription profiles and sphere forming assays of NCCs from *P0-Cre/floxed-EGFP* mouse embryos. We identified up-regulation of genes linked to carcinogenesis in cNCCs that were not previously reported to be related to NCCs, which was considered to be, an interesting feature in regard with carcinogenic potentials of NCCs such as melanoma and neuroblastoma. Wnt signal related genes were statistically up-regulated in cNCCs, also suggesting potential involvement of cNCCs in carcinogenesis. We also noticed intense expression of mesenchymal and neuronal markers in cNCCs and tNCCs, respectively. Consistent results were obtained from *in vitro* sphere-forming and differentiation assays. These results were in accordance with previous notion about differential potentials of cNCCs and tNCCs. We thus propose that sorting NCCs from *P0-Cre/floxed-EGFP* mice might be useful for the basic and translational research of NCCs. Furthermore, these newly-identified genes up-regulated in cNCC would provide helpful information on NC-originating tumors, developmental disorders in NCC derivatives, and potential applications of NCCs in regenerative medicine.

## Introduction

Neural crest cells (NCCs) are cell populations that originate in the early stage of the vertebrate embryo from the dorsal region of the neural tube. They delaminate from the border of neural and non-neural areas of the ectoderm. After delamination, NCCs vigorously proliferate during migration towards various locations within the embryonic body, and differentiate into a wide range of cell types and tissues, including neurons and glial cells of the peripheral nervous systems (PNS), smooth muscles of the heart and great vessels, bone, cartilage, connective tissue of the face, and melanocytes in the skin.

The migration patterns and differentiation fates of NCCs have been well characterized in avian and rodent embryos [1]. Trunk NCCs (tNCCs) emerge from the trunk region of the neural epithelium, and those migrating just beneath the ectoderm will form pigment cells in the skin and others taking a ventral pathway via the somites will differentiate into neurons and glia of the PNS as well as chromaffin cells in the adrenal gland [2]. Craniofacial NCCs (cNCCs) emerge from the forebrain, midbrain and hindbrain regions of the neural epithelium, and populate the frontonasal area or the pharyngeal arches depending on their original positions [3]. These cNCCs produce not only neurons, glia and melanocytes, but also the majority of the connective and skeletal tissue of the head [1]. Therefore, cNCCs show wider variation in their differentiated cell types than tNCCs during normal development.

Another feature characteristic to NCCs is its relation to tumor formation. Melanoma is a common skin cancer derived from pigment cells of NC-origin [4]. It is also believed that neuroblastoma, one of the most frequent child cancers occurred in the sympathetic nervous systems and adrenal gland, is originated from the NCCs [5]. Another example of a cancer thought to be NC-origin is Ewing sarcoma, an aggressive bone and soft tissue tumors [6]. Considering a recent idea of cancer stem cells [7], [8], NCCs may share molecular features common to malignant tumors.

In the present study, we performed transcriptome analyses of cNCCs and tNCCs using genetically engineered mice that specifically label NCCs. We also clarified difference in expression profiles of cNCCs and tNCCs from those of inducible pluriopotent stem cells (iPSCs) and embryonic stem cells (ESCs). Furthermore, we also carried out sphere-forming and differentiation assays to know proliferation and differentiation potentials of cNCCs and tNCCs *in vitro*. Both of approaches consistently revealed differential characters of NCCs as multipotent stem cells, and possibly as cancer stem cells. These results not only provide useful information for NCC application in regenerative medicine but also contribute to develop specific therapeutics for preventing metastatic cascades of NC-derived tumors.

## Materials and Methods

### Animals

Transgenic (TG) mice expressing the Cre enzyme induced by the myelin protein zero (P0) promoter [9] were crossed with the *CAG-CAT-EGFP* TG line [10]. In *P0-Cre/floxed-EGFP* double TG (*P0-Cre; EGFP*) mice, NCCs were identified by evaluating the expression of EGFP after *P0-Cre*-mediated DNA recombination [11]. To eliminate pigmentation in the embryonic tissue of double TG lines, mice originally of the C57/BL6J background were crossed with mice of an ICR background (Japan Charles River, Tokyo, Japan) for 5–6 generations as described previously [12]. At mid-day of identifying a vaginal plug was considered as E0.5. P0-Cre recombinase TG mice were kindly provided by Dr. K. Yamamura (Kumamoto University, Kumamoto, Japan). *CAG-CAT-EGFP* TG mice were kindly provided by Dr. J. Miyazaki (Osaka University, Osaka, Japan) and maintained at Tohoku University. All experimental animal procedures described in this study were approved by the Ethics Committee for Animal Experiments of Tohoku University Graduate School of Medicine (#2012-134).

### Preparation of cells from mouse embryos

*P0-Cre; EGFP* embryos were resected into Hanks' balanced salt solution (HBSS^+^; GIBCO 14025-092) containing 10% fetal bovine serum (FBS; GIBCO 12483) and 1% penicillin/streptomycin (P/S; GIBCO 15140-122). The craniofacial and trunk regions were separated, and heart fields were separated from the trunk region. Each fraction was incubated with 0.25% collagenase (Sigma C5894) in HBSS^+^ for 30 min at 37°C. After rinsing in PBS, embryonic tissues were incubated in 0.25% trypsin-EDTA for 30 min and then mechanically dissociated in HBSS^+^ containing FBS. The cells were collected by centrifugation at 800×*g* for 5 min at 4°C.

### Flow cytometric analysis and fluorescence-activated cell sorting

For flow cytometry and cell sorting, a FACS Aria II (BD Biosciences, San Diego, CA, USA) was used. Sorted EGFP^+^ and EGFP^−^ cells were resuspended in sphere culture medium and cultured in non-adhesive 12-well culture plates (Cell-Seed, Tokyo, Japan CS2018).

### RNA microarray analysis

Total RNA was obtained from harvested EGFP^+^ and EGFP^−^ cells using an RNeasy plus Micro Kit (Qiagen 74034) and DNase I (Qiagen 79254). Comprehensive gene expression microarray analysis was performed with a 3D-Gene (Toray Industries). Data were analyzed using R language [13]. We also analyzed using Gene Spring (Agilent Technologies, Santa Clara, CA, USA). The microarray data has been submitted to the GEO database (accession No: GSE39191).

### Analysis of stem cell gene expression

Stem cell genes with the following GO annotations were selected; “stem cell maintenance” (GO:0019827), “germ-line stem cell maintenance” (GO:0030718), “somatic stem cell maintenance” (GO:0035019), “stem cell differentiation” (GO:0048863), “stem cell development” (GO:0048864), “hemopoietic stem cell differentiation” (GO:0060218). An F-test was applied to evaluate alterations in gene expression of stem cell genes compared with that of all genes on the microarray. Biologically duplicated samples were averaged for the F-test. The Bonferroni multi-test correction was applied to the data.

### Clustering analysis

For gene clustering of the microarray analysis, we first selected 347 genes significantly altered in any of the samples under two criteria; an ANOVA FDR p-value of <0.05 and at least a 4-fold expression change. Then, gene clustering was performed using hclust (complete linkage, 1 - Pearson's correlation) and cutree functions in R language [13]. The number of clusters was manually set to seven. Finally, GO enrichment tests were performed for each cluster using BiNGO software [14]. Because the enriched GO terms for each cluster were numerous, we omitted highly redundant GO terms including more than 80% of genes in a more significant GO term.

### qRT- PCR analysis

Total RNA was obtained by the same method used for microarray analysis as described above. Reverse transcription was performed with a SuperScript First-Strand® III Synthesis Super Mix for qRT-PCR (11752-250, Invitrogen Carlsbad, NM, USA), according to the manufacturer's instructions. qRT- PCR was performed using an ABI Prism 7900HT Sequence Detection System (Applied Biosystems Inc., Foster City, CA, USA), according to the manufacturer's instructions. Primer pairs and TaqMan® MGB probes were designed with Assay by-Design™ (Applied Biosystems). Results were evaluated using the Student's *t* test.

### Immunohistochemistry

Cultured cells and frozen tissue sections were fixed with 4% paraformaldehyde and then stained with the following primary antibodies: anti-Sox10 (N-20; Santa Cruz Biotechnology, Santa Cruz, CA, USA), anti-PDGF receptor α-chain (558774; BD Pharmingen, San Diego, CA, USA), anti-PDGF receptor β-chain (#3169; Cell Signaling Technology, Danvers, MA, USA), anti-GFP (ab13970; Abcam, Cambridge, UK), anti-Ap2α, anti-AP-2β (Cell Signaling Technology), anti-Nanog (AB5731; Millipore, Temecula, CA, USA) anti-Nestin (G-20; Santa Cruz Biotechnology), anti-Oct3/4 (MAB1759; R&D Systems, Minneapolis, MN, USA), anti-PAR4 (3G9H7; Santa Cruz Biotechnology), anti-Sox2 (Y-17; Santa Cruz Biotechnology), anti-alpha smooth muscle actin (ab32575; Abcam), anti-βIII-tubulin (MAB1195; R&D Systems), anti-glial fibrillary acidic protein (MAB360; Chemicon International, Temecula, CA, USA) and anti-GFP (MBL, Nagoya, Japan). After washing with Tris-buffered saline, the sections were stained with Alexa Fluor-488 or Alexa Fluor-568 conjugated secondary antibodies (Invitrogen). All sections were counterstained with Hoechst 33342 (Invitrogen).

### Cell culture

Sorted EGFP^+^ and EGFP^−^ cells were cultured in DMEM/F-12 (1∶1) supplemented with 20 ng/mlL epidermal growth factor (EGF; Sigma-Aldrich), 20 ng/ml basic fibroblast growth factor (bFGF; Invitrogen), B27 supplement (Invitrogen) and 10^3^ units/ml leukemia inhibitory factor (LIF; Chemicon) at 37°C with 5% CO_2_. LIF was added to prevent differentiation as previously described [12]. Cells were seeded onto non-adhesive 6-well culture plates (Cell-Seed) and cultured for 5–7 DIV. To obtain secondary spheres, primary spheres were dissociated with 0.05% trypsin 0.53 mM EDTA-4Na (Invitrogen, Carlsbad, NM, USA) into a single-cell suspension and then re-seeded into fresh medium.

### Differentiation culture

To induce neurogenic and glial differentiation, EGFP^+^ and EGFP^−^ spheres were cultured in sphere culture medium without bFGF, EGF or LIF for 7 DIV on dishes coated with ornithine and laminin. For chondrogenic differentiation, spheres were cultured using a hMSC Differentiation Bulletkit, Chondrogenic (Lonza) according to the manufacturer's instructions, and evaluated by staining with alcian blue (Diagnostic Biosystems, Pleasanton, CA, USA). Smooth muscle differentiation was induced by culture in DMEM/F12 containing 2% FBS and 10 ng/ml transforming growth factor β1 (R&D Systems) for 7 DIV. For quantification of adipocyte differentiation by relative fluorescence units (RFU), excitation of the Adipo Red-stained cells was measured at excitation and emission at 485 and 535 nm, respectively.

## Results

### Isolation of NCCs from *P0-Cre/Floxed-EGFP* mouse embryos

To elucidate the molecular features of NCCs, we planned to isolate NCCs from mouse embryos that contain genetically labeled NCCs. Wnt1-Cre is widely used among NCC-specific Cre-driver lines [15], but this line shows strong activity of Cre in the midbrain region [16]. Another line, i.e., P0-Cre has been established by utilizing a promoter of a gene encoding Shwann cell-specific P0 protein. The specificity of P0-Cre activity in various mouse tissues, especially craniofacial region, has been well proven by checking lack of expression of Cre mRNA and protein [11], [12], [17]–[19] except for a very minor leaky labeling in the mesoderm-derived notochord [9], Therefore, we chose *P0-Cre/Floxed-EGFP* mice to analyze molecular and cellular characters of NCCs.

**Figure 1:**
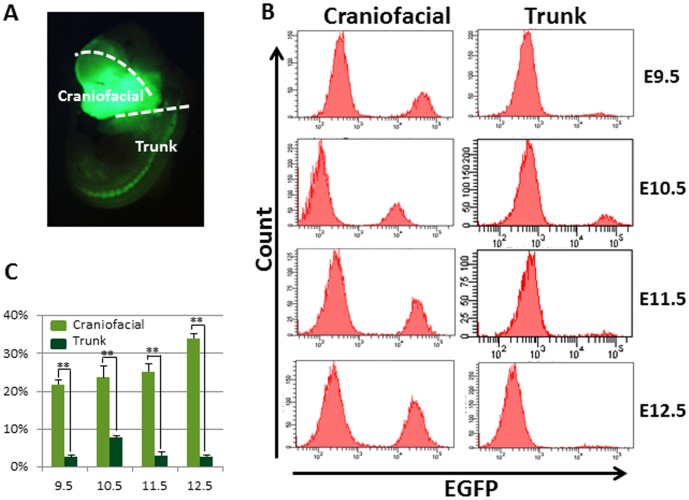
Isolation of NCCs from *P0-Cre/Floxed-EGFP* mouse embryos by fluorescence-activated cell sorting. (A) Craniofacial and trunk regions were indicated in the whole body by observation of direct EGFP fluorescence in E12.5 mice. (B) Representative EGFP-gated flow cytometric analysis charts clearly showed two populations, EGFP positive and negative at all examined embryonic ages. (C) The ratio of collected EGFP^+^ cells showed a significantly higher frequency in the craniofacial region than that in the trunk region of all examined embryonic ages. Results were evaluated using the Student's *t*-test. (mean ± SD, n = 5 per group, **p<0.005).

To isolate cNCC and tNCCs, craniofacial and trunk regions were separately dissected from *P0-Cre/Floxed-EGFP* mouse embryos at embryonic day (E) 9.5, E10.5, E11.5 and E12.5 (Fig. 1A). Cells were dissociated from the both regions and analyzed by flow cytometry to assess the intensity of EGFP. These cells were separated into two populations, P0-EGFP positive (EGFP^+^) and negative (EGFP^−^) cells (Fig. 1B). The percentage of EGFP^+^ cells from the craniofacial region increased with embryonic age, while in the trunk region, the frequency of EGFP^+^ cells was relatively lower than that in the craniofacial region with its highest ratio at E10.5 (Fig. 1C). Therefore, although we noticed that these EGFP^+^ cells might contain heterogeneous cell populations, we further analyzed these EGFP^+^ and EGFP^−^ cell populations from craniofacial and trunk regions.

### Expression of typical NCC markers by quantitative real-time (qRT)-PCR

**Figure 2:**
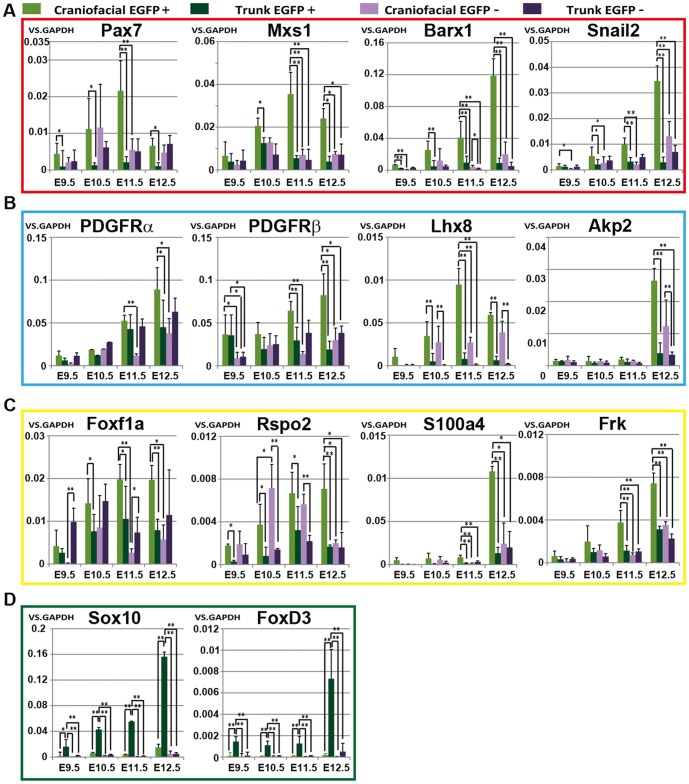
Quantitative PCR analysis of selected genes based on transcriptome analyses. EGFP^+^ and EGFP^−^ cells obtained from craniofacial and trunk regions at E9.5, E10.5, E11.5 and E12.5 were examined. (A) NCC markers up-regulated in cNCCs (Pax7, Msx1, Barx1 and snail2). (B) Mesenchymal markers up-regulated in cNCCs (PDGFRα, PDGFRβ, Lhx8 and Akp2. (C) Candidate markers up-regulated in cNCCs (Foxf1a, Rspo2, S100a4 and Frk). (D) NCC markers up-regulated in tNCCs (Sox10 and FoxD3). (mean±SD, n = 5 per group, *p<0.05, **p<0.005).

To evaluate NCC character in EGFP^+^ cells, we examined expression of several NCC and/or mesenchymal markers by qRT-PCR analysis in EGFP^+^ and EGFP^−^ cells from craniofacial and trunk regions in E9.5, E10.5, E11.5 and E12.5 embryos. Frequently used markers of NCCs *Pax7*, *Msx1*, *Barx1*, *snail2* and *PDGFRα* showed higher expression in craniofacial EGFP^+^ cells (Cp) at all embryonic stages examined (Fig. 2A). As we expected, mesenchymal markers such as *Lhx8* and *Akp2* also showed higher expression in Cp (Fig. 2B). *PDGFRβ*, a well-known marker of pericytes, exhibited the same tendency (Fig. 2B). We noticed strong expression of these genes in Cp from E12.5 embryos, while other NCC markers such as *FoxD3 and Sox10* showed remarkably higher expression in trunk EGFP^+^ cells (Tp) than in Cp at all stages, especially at E12.5 (Fig. 2D). Therefore, we further analyzed global gene expression profile in E12.5 embryos even though they possibly included already-differentiating NCCs.

### Expression profiles of cNCCs and tNCCs include genes related to carcinogenesis

**Figure 3:**
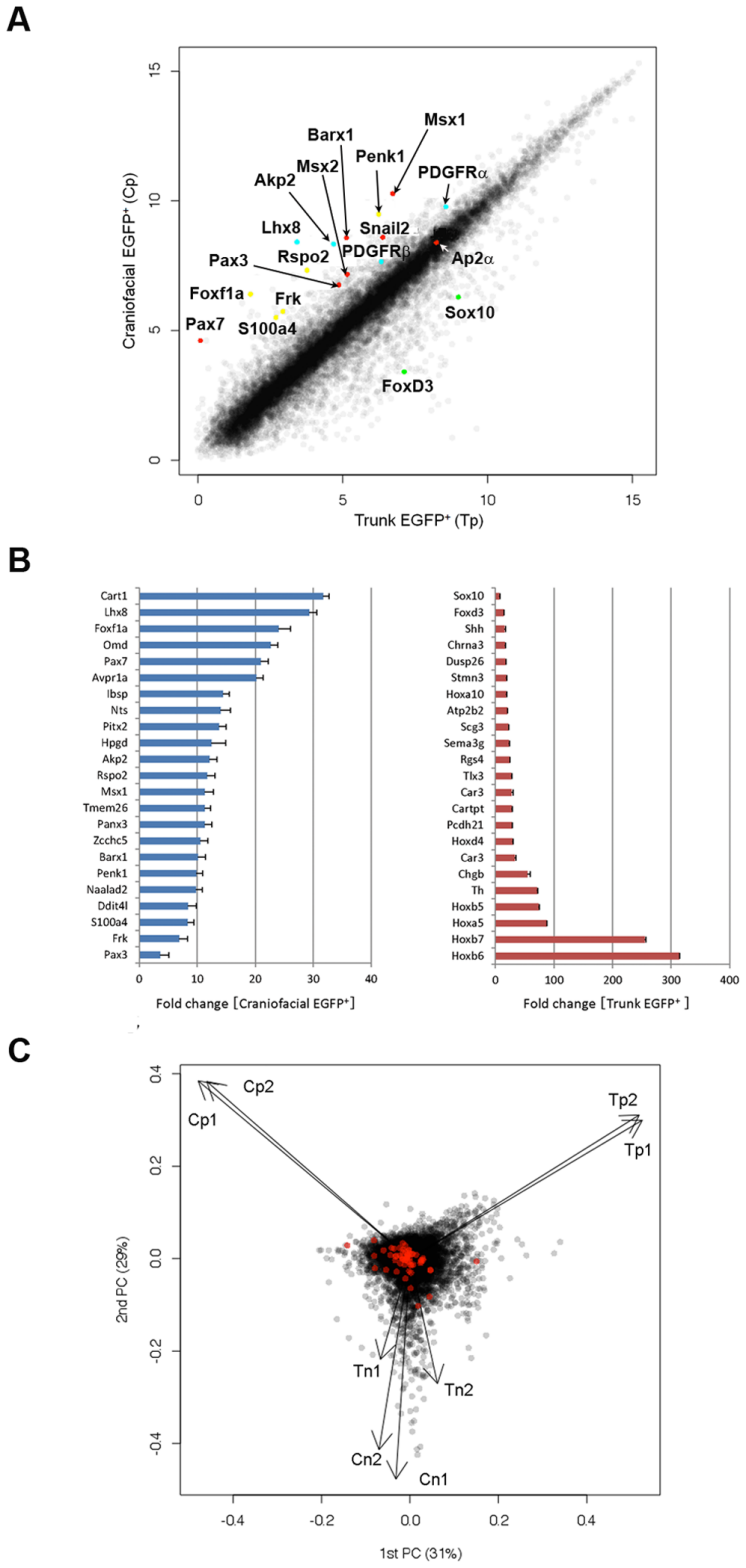
Differential expression profiles of cNCCs and tNCCs in *P0-Cre/Floxed-EGFP* mouse embryos. (A) Scatter plot of Craniofacial EGFP^+^cells (Cp) and Trunk EGFP^+^cells (Tp) as assessed by microarray analysis (3D-Gene; Toray Industries). (B) Most up-regulated genes in Craniofacial EGFP^+^ cells (blue) and Trunk EGFP^+^ cells (red), compared with those in the EGFP^+^ cells of trunk and craniofacial regions, respectively. (C) Biplot of principal component analysis of the eight samples revealed three sample groups. Black dots indicate all genes and red dots indicate known stem cell genes selected from GO annotations. Cp, Tp, Cn; craniofacial EGFP^−^ cells, and Tn; trunk EGFP^−^ cells.

To identity differences in the global mRNA profiles of Cp versus Tp, and EGFP^+^ versus EGFP^−^ cells, we performed transcriptome analyses using 3D-Gene (Toray Industries) of biologically duplicated samples from embryos at E12.5 when NCCs proliferate and start to differentiate. Representative scatter plots indicated differential molecular features of cNCCs and tNCCs (Fig. 3A). Compared with Tp, transcripts more enriched in Cp included molecules known as mesenchymal markers (*Cart1*, 31-fold; *Lhx8*, 29-fold; *Omd*, 23-fold; *Ibsp*, 14-fold; and *Akp2*, 12-fold) (Fig. 3B, left). This may reflect character of cNCCs giving rise to bone, cartilage, teeth, and connective tissues. NCC markers (*Pax7*, 21-fold; *Msx1*, 11-fold; *Barx1*, 10-fold; *Pax3*, 3.6-fold; and *PDGFRα* 2.8-fold) showed higher expression in cNCCs than in tNCCs (Fig. 3B, left). Unexpectedly, we identified several genes that have not previously been reported in association with NCCs but are suggested to be related with carcinogenesis. These genes showed higher expression in Cp than that in Tp (*Foxf1a*, 24-fold; *Rspo2*, 12-fold; *Penk1*, 10-fold; *S100a4*, 8-fold; and *Frk1*, 7-fold). Among these genes, we further confirmed the actual expression of the four genes (*Foxf1a*, *Rspo2*, *S100a4*, *Frk*) by qRT-PCR (Fig. 2C). These genes up-regulated in cNCC might provide a new insight for NC-originating tumorigenesis (see discussion).

Genes showing higher expression in Tp than that in Cp included a few well-known NCC markers, *Foxd3* (13-fold) and *Sox10* (6.7-fold) (Fig. 3B, right). In addition, several *Hox* genes showed remarkably higher expression in Tp, compared with that in Cp (*Hoxb6*, 313-fold; *Hoxb7*, 254-fold; *Hoxa5*, 87-fold; *Hoxb5*, 74-fold; *and Hoxd4*, 24-fold) (Fig. 3B, right); these *Hox* genes were also highly expressed in trunk EGFP^−^ cells (Tn; Table S1).

We applied principal component analysis to summarize the transcriptome analyses for the four samples with biological duplication. On the biplot of the analysis, genes and samples are shown as points and vectors, respectively (Fig. 3C). The first and second principal components (PCs) clearly showed three distinctive sample groups, namely Cp, Tp, and the others (craniofacial EGFP^−^ cells; Cn and Tn). Among the three sample groups, the Cp and Tp sample groups were particularly reproducible with biological duplicates, indicating that Cp and Tp sample groups clearly represented an NCC lineage.

To investigate the stem cell-like character of these samples, we selected 60 genes with known functions relating to stem cells (termed here as “stem cell genes”) based on Gene Ontology (GO) annotations, and overlaid them on the biplot of the PC analysis (red points in Fig. 3C). Interestingly, most stem cell genes distributed along the Cp vector. We confirmed a large alteration in the expression of stem cell genes in Cp by applying an F-test to variances of the stem cell genes against all the genes on the microarray. The alteration of the stem cell genes was significantly larger than that of all genes in the Cp sample (p = 0.0008), while the alteration in the other three samples was not significant (p = 1). These results are considered to reflect the stem cell-like character in gene regulation of cNCCs.

We also performed clustering analyses of the four samples. Differentially expressed genes in Cp, Tp, Cn and Tn revealed considerable variation, which yielded seven probe clusters that showed a specific gene expression pattern for each sample (Fig. S1, Table S1). GO enrichment analyses of the clusters revealed that most of the genes specifically expressed in the four groups were in functional categories related to regulation of differentiation, development, and morphogenesis (see Tables S2, S3, S4, S5, S6, S7 for reference). Genes related to “Wnt signal” and “development” were highly enriched in Cp (Fig. S1A, Table S2). On the other hand, a category related to “nervous system development” was over-represented by up-regulated genes in Tp (Fig. S1C, Table S3). Genes related to “forebrain development” were up-regulated in Cn (Fig. S1D, Table S4). In addition, a category related to “anterior posterior pattern formation” was over-represented by up-regulated genes in both Tp and Tn (Fig. S1G, Table S7). These results indicate regional differences in the expression of developmental genes, particularly in EGFP^+^ cells. In summary, cNCCs and tNCCs regions are extensively featured with their gene expression profiles that reflect their differential differentiation state and potential.

### Differential gene expression profiles between NCCs and pluripotent stem cells

**Figure 4:**
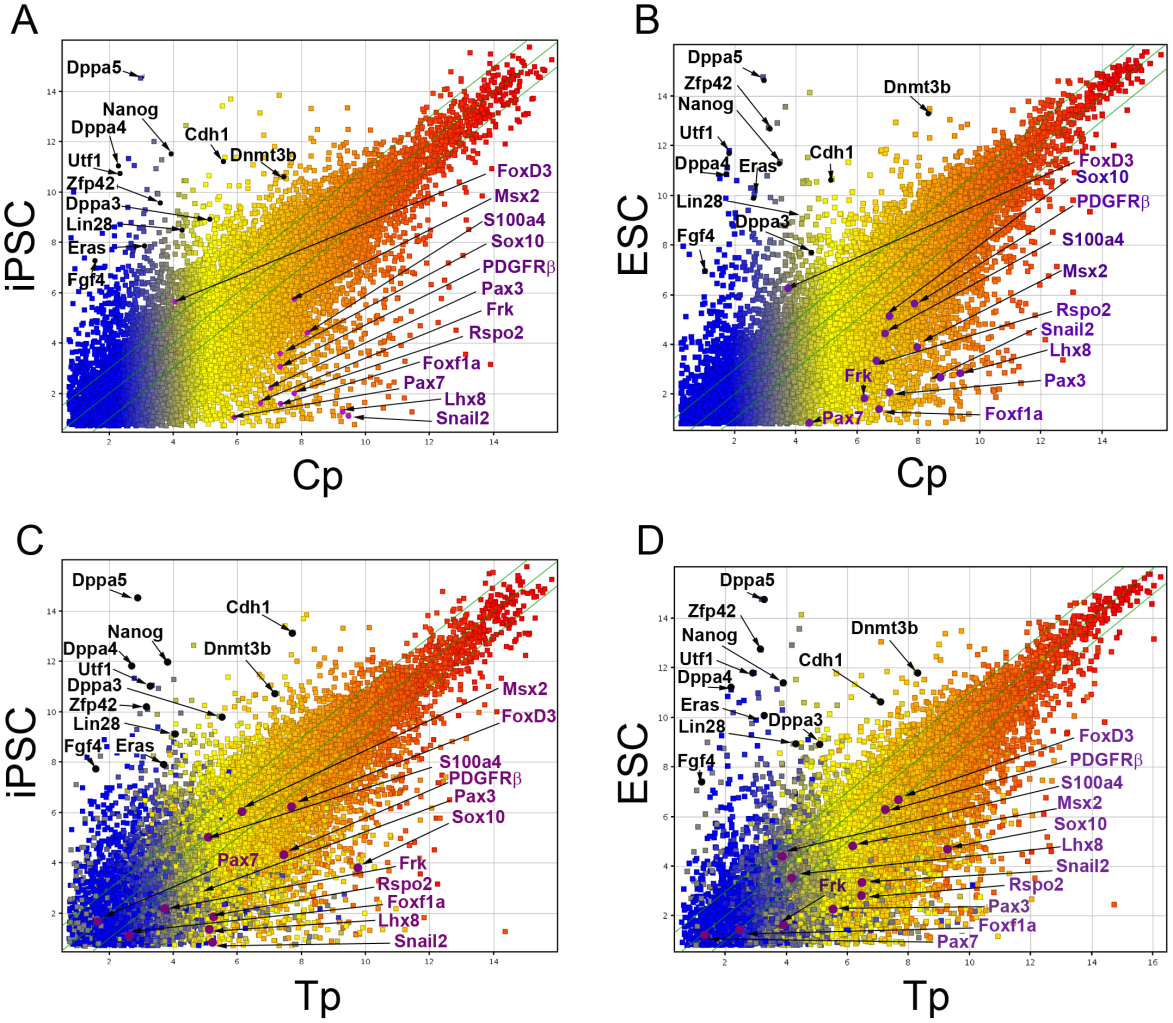
The comparison of gene expression analysis of Cp and/or Tp versus pluripotent stem cells. Schatter plot of Cp and iPSCs (A), Cp and ESCs (B), Tp and iPSCs (C), Tp and ESCs. Pluripotent markers were indicated by block charcters. NCC markers were indicated by violet characters.

To estimate pluripotency of cNCCs and tNCCs, we examined the similarities of their gene expression to those of mouse iPSCs and ESCs cited from GEO database. Since both iPSC and ESC experiments (GEO accession: GSE18117 and GSE18813, respectively) used the same microarray platform with ours, we directly compared expression levels of genes on this platform. The scatter plot of the average of duplicate samples of Cp and Tp versus iPSCs or ESCs showed that both of the cNCCs and tNCCs similarly expressed lower levels of the well-known pluripotential markers (Fig. 4A–D). The expression levels of these markers were quite low in Cn and Tn (Data not shown). In contrast, the levels of NCC marker genes except for *FoxD3*, were lower in iPSCs and ESCs than in NCCs (Fig. 4A–D). In spite of significant alteration in expression of stem cell genes (Fig. 3C), the pluripotent marker genes showed similarly weak expression in Cp, Tp, Cn and Tn (Fig. 4A–D).

### Confirmation of NCC-specificity in *P0-Cre/Floxed-EGFP* mouse embryo by immnohistchemistry

The expression patterns of the genes showing differential mRNA levels were further examined at the protein level by immunohistochemistry. For example, PDGFRα and PDGFRβ proteins were detected in craniofacial mesenchymal cells expressing EGFP, while Sox10 protein was selectively expressed in EGFP^+^ cells located in the dorsal root ganglia (DRG), a major NC derivative (Fig. S2). Considering the other staining results (Fig. S3), the majority of cNCCs showed the molecular character of mesenchymal derivatives, while the majority of tNCCs exhibited that of neural derivatives. These findings of the molecular features of NCCs are consistent with their differential states or fates as previously suggested (see Discussion). This again confirmed that EGFP^+^ cells in *P0-Cre/floxed-EGFP* mouse reflect their origin as NC-derived.

### Sphere-forming capability of cNCCs and tNCCs

**Figure 5:**
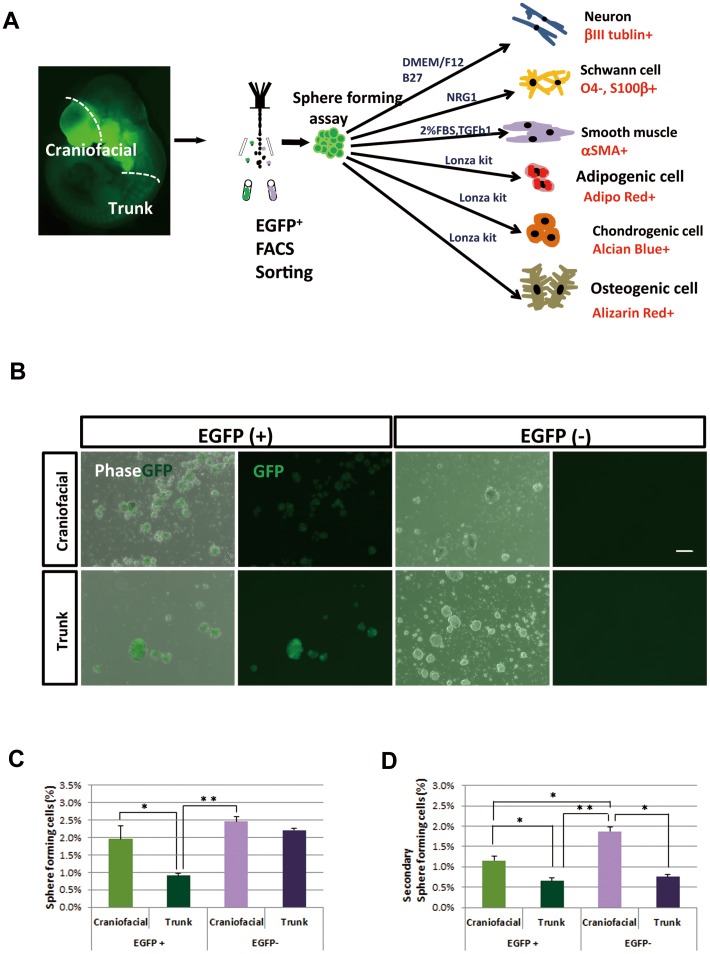
Sphere-forming capacity of EGFP^+^ and EGFP^−^ cells from craniofacial and trunk regions. (A) Schematic illustration of the experimental design for isolation and differentiation of P0-EGFP^+^ cells from *P0-Cre/Floxed-EGFP* mouse embryos at E12.5. (B) Phase-contrast and direct EGFP fluorescence images showing spheres formed by EGFP^+^ and EGFP^−^ cells derived from craniofacial and trunk regions, respectively, after 5 DIV. Scale bar, 50 µm. (C, D) The percentage of sphere-forming cells assessed by culturing EGFP^+^ and EGFP^−^ cells from each region at a cell density of 5×10^3^ cells/ml and counting the number of formed spheres. (mean ± SD; n = 5 per group, *p<0.05,**p<0.005). A significantly higher frequency of primary spheres (C) and secondary spheres (D) were formed by craniofacial EGFP^+^ cells, compared with those formed by trunk EGFP^+^ cells.

We next assessed the stem cell-like characters of cNCCs and tNCCs by sphere-forming assays (Fig. 5). EGFP^+^ and EGFP^−^ cells dissociated from the craniofacial and trunk regions in E12.5 *P0-Cre/Floxed-EGFP* embryos were isolated by fluorescence-activated cell sorting, and cultured at 5×10^3^ cells/ml in a serum-free sphere-forming medium containing growth factors, leukemia inhibitory factor (LIF) and B27 supplement as described previously [12]. Both EGFP^+^ and EGFP^−^ cell populations obtained from the two regions proliferated and formed spheres (Fig. 5B, C). This is quite contrast with the fact that few spheres are formed by adult EGFP^−^ cells from the DRG, skin, whisker pad and bone marrow of *P0-Cre/Floxed-EGFP* mice [19]. We do not know whether this discrepancy is due to our different culture conditons (with LIF to enhance immature state or without) or to different stages of tissues (adult or embryonic). Among all four groups, the highest number of spheres was formed by craniofacial EGFP^−^ cells (Fig. 5C). This may be because EGFP^−^ cells contained neural stem cells located within the neural tube. Comparing regional difference of NCCs, a higher number of spheres were formed by craniofacial EGFP^+^ cells than that by trunk EGFP^+^ cells (Fig. 5C).

To assess the self-renewal capacity of these spheres, we conducted secondary sphere-forming assays. EGFP^+^ and EGFP^−^ spheres derived from both regions of *P0-Cre/Floxed-EGFP* mouse embryos were dissociated into single cells, and then cultured at the same density as that for primary spheres in sphere-forming medium. Secondary spheres were formed by all cells from primary spheres with a highest frequency in craniofacial EGFP^−^ cells (Fig. 5D). Comparing regional difference, craniofacial EGFP^+^ cells showed a higher level of secondary sphere formation than trunk EGFP^+^ cells (Fig. 5D). This suggests that cNCCs have a higher capacity for self-renewal than that of tNCCs.

**Figure 6:**
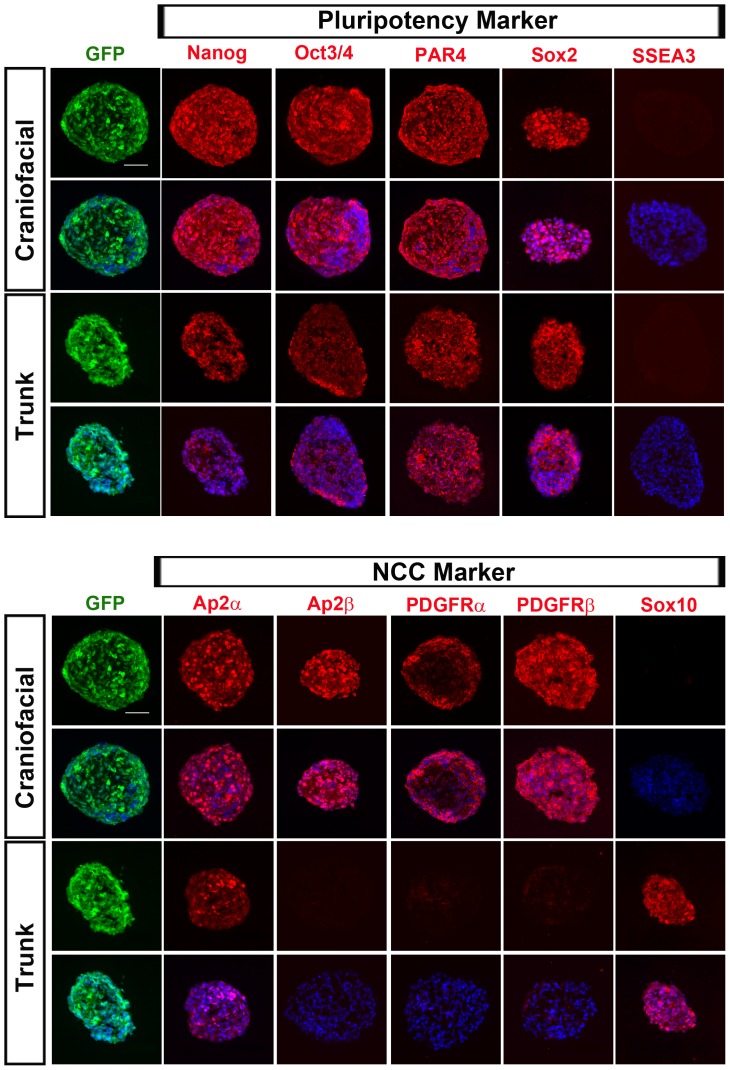
Immunocytochemical analyses of spheres derived from the EGFP^+^ cells of craniofacial and trunk regions. (A) Expression of pluripotency markers (Nanog, Oct3/4, PAR4 and Sox2), except SSEA3, in EGFP^+^ spheres derived from both regions. (B) Differential expression of NCC markers (Ap2α, Ap2β, PDGFRα, PDGFRβ and Sox10) in spheres between craniofacial and trunk EGFP^+^ cells (scale bar; 20 µm).

Next, we examined the molecular features of spheres cultured for 5 days *in vitro* (DIV). As expected, spheres formed by EGFP^+^ cells highly expressed pluripotency markers Nanog, Oct3/4, PAR-4 and Sox2, with the exception of SSEA3 (Fig. 6). These spheres were also positive for NCC markers Ap2α, Ap2β, PDGFRα PDGFRβ and Sox10 (Fig. 6). The difference in expression levels of NCC markers in the spheres formed by craniofacial versus trunk EGFP^+^ cells (Fig. 6) was in accordance with qRT-PCR and immunohistochemistry data of the embryos (Fig. 3 and S2). Expression of mesenchymal and NCC markers, *PDGFRα* and *PDGFRβ* was observed predominantly in spheres formed by craniofacial EGFP^+^ cells, whereas *Sox10* expression was prominent in spheres formed by trunk EGFP^+^ cells (Fig. 6). These results indicate that cultured spheres reproduce the differential features of cNCCs and tNCCs similarly to that *in vivo*.

### Spheres derived from NCCs produce multiple cell types

**Figure 7:**
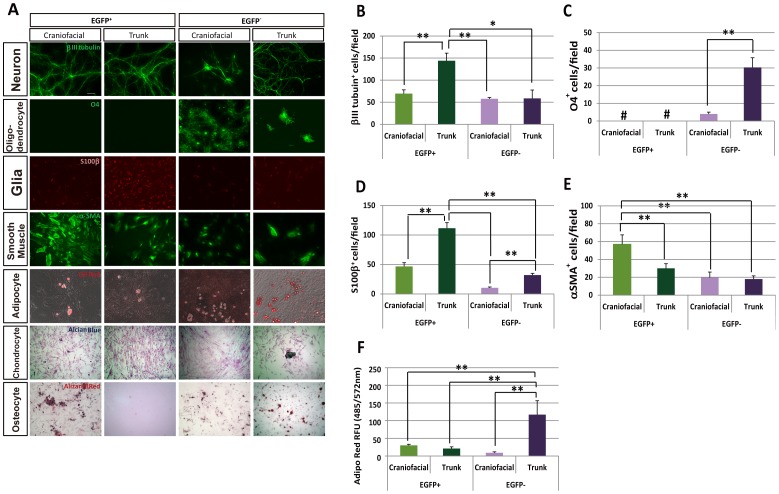
Differentiation potential of spheres derived from EGFP^+^ and EGFP^−^ cells from craniofacial and trunk regions. (A) Broad range of differentiation potential in spheres derived from craniofacial EGFP^+^ cells. All groups of spheres differentiated into neurons, glial cells, myofibroblasts and adipocytes. Chondrocytes were differentiated from only spheres derived from craniofacial EGFP^+^ cells and trunk EGFP^−^ cells. Osteocytes were differentiated from all groups of spheres except for spheres derived from trunk EGFP^+^ cells. (B–F) Spheres derived from trunk EGFP^+^ cells showed peripheral neuronal lineages. Quantitative analyses of the potential for differentiation into neurons (B), oligodendrocytes (C), glial cells including Schwann cells (D), and myofibroblasts (E) by counting the number of cells positive for specific markers. (F) Quantification of adipocyte differentiation by relative fluorescence units (RFU). Excitation and emission of Adipo Red-stained cells was measured at 485 and 535 nm, respectively. Trunk EGFP^−^ cells showed the highest differentiation potential among the four groups.

To evaluate the differentiation potential of spheres, we cultured EGFP^+^ and EGFP^−^ spheres, obtained from the craniofacial and trunk regions of *P0-Cre/Floxed-EGFP* mouse embryos, in various differentiation media suitable for inducing individual tissue types (Fig. 7A). The four populations showed overlapping yet differential phenotypes in differentiating potentials.

Neuronal differentiation was induced by withdrawal of fibroblast growth factor 2 (FGF2), epidermal growth factor (EGF) and LIF from the medium. All four types of spheres frequently yielded neurons marked with βIII-tubulin (Fig. 7A), but a greater number of neurons were observed in spheres derived from the trunk EGFP^+^ cells (Fig. 7B; 144 cells/field). The frequency of neurons in the other three types of spheres was almost identical (Fig. 7B; 69.7 cells/field for craniofacial EGFP^+^; 58.0 cells/field for craniofacial EGFP^−^; 58.5 cells/field for trunk EGFP^−^). These results indicated that trunk EGFP^+^ cells contained larger number of neuronal lineage NCCs than craniofacial EGFP^+^ cells.

Glial differentiation was induced by addition of neuregulin (Fig. 7A, C, D). Only spheres derived from craniofacial and trunk EGFP^−^ cells yielded O4-positive oligodendrocytes with a dramatically larger amount from the trunk EGFP^−^ cells (Fig. 7C; four cells/field for craniofacial, and 30 cells/fields for trunk). S100β-positive glial cells including Schwann cells were obtained from all four types of spheres, with the greatest number in those derived from trunk EGFP^+^ cells (Fig. 7D; 46.6 cells/fields for craniofacial EGFP^+^; 111.7 cells/fields for trunk EGFP^+^; 10.3 cells/fields for craniofacial EGFP^−^; and 32 cells/fields for trunk EGFP^−^). This quantification of neuronal and glial cell differentiation clearly showed that EGFP^+^ spheres derived from the trunk region produced the highest frequency of neural lineages.

We further examined the potential to differentiate into mesenchymal lineages. EGFP^+^ cells from craniofacial regions were capable of generating myofibroblastic (α-smooth muscle actin^+^), adipogenic (oil red O^+^), chondrogenic (alcian blue^+^), and osteogenic (alizarin red^+^) cells (Fig. 7A). Quantitative analyses were done in cases of myofibroblastic and adipogenic differentiation. EGFP^+^ spheres derived from the craniofacial region showed the highest frequency of differentiation into myofibroblasts (Fig. 7E; 57 cells/fields for craniofacial EGFP^+^; 30 cells/fields for trunk EGFP^+^; 19.7 cells/fields for craniofacial EGFP^−^; and 18 cells/fields for trunk EGFP^−^), while trunk EGFP^−^ cells exhibited the greatest number of adipocytes (Fig. 7F; 30.4 RFU for craniofacial EGFP^+^; 21.0 RFU for trunk EGFP^+^; 9.3RFU for craniofacial EGFP^−^; and 117.2 RFU for trunk EGFP^−^). This observation was expected because mesenchymal derivatives, such as adipocytes, can be differentiated from not only NCCs, but also the mesodermal cells that were observed among EGFP^−^ cells, as speculated from a previous study showing mesodermal stem cells in P0-Cre/YFP mouse [20]. Qualitatively, chondrocytes differentiation was obvious in craniofacial EGFP^+^ cells and trunk EGFP^−^ cells, but not in other two populations (Fig. 7A). Osteocytes were differentiated from all three cell populations, except trunk EGFP^+^ cells (Fig. 7A).

Taken altogether, NCCs purified as EGFP^+^ cells from *P0-Cre/Floxed-EGFP* mouse embryos have a robust capacity for multipotent differentiation. It is of note that cNCCs have the broader differentiation potency for both neural and mesenchymal lineages than tNCCs.

## Discussion

The expression and function of a limited number of NCC-specific molecules have been reported in previous studies [20]–[29]. Here, we identified differential features of cNCCs versus tNCCs at molecular and cellular levels in a systematic manner by combining flow cytometry, transcriptome analyses, sphere-forming assays, and differentiation assays, by the use of *P0-Cre/Floxed-EGFP* mouse embryos. Moreover, we identified a distinction in gene expression profiles of NCCs and pluripotent stem cells. Furthermore, we detected in NCCs up-regulation of genes that are related to carcinogenesis but have not previously been associated with NCCs. Our findings confirm specificity of P0-Cre-labeling of NC-derived cells and provide new insight on tumorigenesis originated from the NC.

Regarding regional difference in molecular and cellular characters of NCCs, we clearly identified genes enriched in cNCCs, namely *Pax7*, *Msx1*, *Barx1*, *Pax3*, *Msx2*, *snail2 and PDGFRα*. These molecules are known to be involved in the development of cNCCs [21], [24]–[29]. This result in turn confirms that we could isolate NCCs or NC-derived immature cells as EGFP^+^ cells from the craniofacial region of *P0-Cre/Floxed-EGFP* mouse embryos.

We also found several molecules that are known as mesenchymal (*Lhx8*), chondrogenic (*Cart1*), and osteogenic (*osteomodulin*, *Akp2*, and *Ibsp*) markers, with prominently higher expression in cNCCs than that in tNCCs. This result was reproduced in GO enrichment analysis. These molecular features are consistent with previous results obtained from transplantation experiments and clonal culture analyses that show the majority of cNCCs differentiate into mesenchymal derivatives [30]–[32].

In addition, Wnt signal-related genes are highly enriched in cNCCs as shown by GO enrichment analysis. Wnt signaling has been reported to play important roles in NC development. NC-delivered cranial structures and dorsal-root ganglion, but not sympathetic ganglion, were defective in *Wnt-1* and *Wnt-3b* null mice and *Wnt-1* and *Wnt-3b-*mediated *β-catenin* conditional knockout mice [33]–[35]. Especially craniofacial defects were strong in the later mice [33]. Thus, our findings further confirm that Wnt signaling is critical for the development of cNCCs.

The majority of tNCCs divergently differentiate into neuronal and glial derivatives [36]. This property is actually reflected in our GO enrichment analyses; *FoxD3* and *Sox10* showed relatively higher expression in tNCCs than that in cNCCs in microarray and also in qRT-PCR analyses. The expression of *FoxD3* is required for NCC multipotency, selectively maintained in neural derivatives, and downregulated in mesenchymal derivatives [23]. *FoxD3* is also expressed considerably in pluripotent stem cells [37]. Mice carrying a mutation in *Sox10* demonstrate deformation in peripheral glia cells of NCC derivatives [38]. Expression of Sox10 continues in the glial lineages in the mouse embryo [39]. Thus, a high level of *Sox10* expression in tNCCs may reflect this situation. Taken together, our transcriptome analyses further confirmed distinct characters of tNCCs and cNCCs at molecular levels.

The stem cell-like character of NCCs has previously been studied *in vitro* using quail embryos [40]. Expression levels of pluripotent markers in cNCCs and tNCCs were weaker than those of iPSCs or ESCs. Similarly, NSCs taken from brain also show weaker expression of the pluripotent markers [41].

By sphere-forming assays, the tendency of primary sphere formation was relatively higher in cNCCs, craniofacial non-NCCs and trunk non-NCCs, and secondary sphere formation was the highest in the craniofacial non-NCCs. This tendency was also true for tertiary spheres. The higher frequency of sphere formation by craniofacial non-NCCs may be because they include neural progenitor cells from the forebrain. We observed robust expression of pluripotency markers and NCC markers in spheres derived from cNCCs and tNCCs. Interestingly, cNCCs showed a higher capacity for self-renewal than that of tNCCs. Significant alteration in the expression of stem cell genes in cNCCs also indicates the self-renewal potential of this tissue. Furthermore, as implicated by the differential molecular features, cNCCs and tNCCs showed a clear difference in terms of the cell types produced during differentiation assays. cNCCs differentiated into mesenchymal lineages such as osteocytes and chondrocytes, whereas tNCCs did not differentiate into either tissue. These results are in accordance with previous reports [42], [43]. In our study, both cNCCs and tNCCs differentiated into adipocytes, as shown in a previous study [44], although trunk EGFP^−^ cells produced the highest number of adipocytes. This result is quite reasonable, because trunk EGFP^−^ cells include a relatively large proportion of mesodermal stem cells that can differentiate into osteocytes, chondrocytes and adipocytes. Regarding neural lineages, the highest number of neuronal cells and Schwann cells were differentiated from tNCCs. This is consistent with a previous report that the majority of tNCCs differentiate into neurons of the PNS, such as sympathetic, parasympathetic and sensory neurons, and Schwann cells [45]. Taken together, our comprehensive study clearly demonstrates that EGFP^+^ cells isolated from *P0-Cre/floxed-EGFP* mouse at mid gestation show NCC features with accuracy and consistency and share a part of the stem cell-like character.

The stem cell-like character of NCCs prompts us to use NCCs that reside within adult tissues as a cell source for autologous transplantation in regenerative medicine. NCCs have been isolated from adult mouse heart [46], craniofacial tissues such as the mouse cornea [47] and iris [12], intestine [48] and from the human dental pulp [49], and evaluated for their properties as stem cells. Using *P0-Cre/Floxed-EGFP* mice would thus be beneficial for not only understanding craniofacial development and disorders, but also for potential resource of cells to induce multiple cell types needed for regenerative therapies.

The third aspect in our study is that we molecularly identified potential involvement of NCCs in tumorigenesis. As already mentioned above, activating Wnt signaling is one of the major mechanisms in formation of cancer [50]. We also identified up-regulation of cancer-related genes in NCCs. For example, *Frk*, a member of the small family of intracellular Src-related tyrosine kinases and a potential tumor suppressor [51], was originally identified in a human hepatoma cell line [52], and is expressed predominantly in epithelial tissues [49]. *S100a4*, a member of the S100 family of calcium-binding proteins, has been shown to activate pathways characteristic of cancer metastasis [53]. Given that NCCs share the stem cell-like potentials, expression of these cancer-related genes in NCCs may imply that NCCs can generally play important roles in carcinogenesis. Further analyses are required to examine potential up-regulation of these cancer-related genes in NCCs.

Involvement of NCCs in formation of tumors is intriguing. Melanoma is obviously derived from the NC-derived pigment cells in the skin [54]. Neuroblastoma and pheochromocytoma are formed in the adrenal gland and/or sympathetic ganglia, and thus believed to be NC-origin [55]. Our findings that NCCs show molecular features related with tumorigenesis might further suggest involvement of NCCs in other cancers. For example, origin of glioma is still enigmatic. A recent report has suggested oligodendrocyte precursor cells (OPCs) may participate in formation of glioma [56]. In this study, glioma has efficiently been induced by conditional genetic engineering using OPC-specific driver, NG2-Cre. We have recently reported in *P0-Cre/EGFP* mouse that NG2 is specifically expressed in cNCCs in the embryonic forebrain [17]. The majority of NCCs differentiated into the pericytes, but it seems that a small population of NCCs remains in an undifferentiated state [17]. The expression of NG2 in NCCs in the brain could thus provide insight that NG2-driven glioma formation might be derived also from NCCs. Furthermore, OPCs and pericytes express PDGFRα [57] and PDGFRβ [58], respectively, and it has been shown that PDGF is a strong inducer of glioma [59]. Our studies and previous reports have also shown expression of PDGF receptors in NCCs [17], [20], [60]. Therefore, it would be worth to try whether glioma can be induced by specifically targeting cNCCs. Future study on understanding NCCs might also contribute to prevent various tumors of NC-origin.

## Supporting Information

Figure S1 **Clustering on the data sets from general filtering of four populations.** From these four populations (Cp, Tp, Cn, Tn), we identified 7 probe clusters. (A) Cp up-regulation (B) Cp down-regulation (C) Tp up-regulation (D) Cn up-regulation (E) Cp and Tp up-regulation (F) Cn and Tn up-regulation (G) Tp and Tn up-regulation.
(TIF)

Figure S2 **Expression patterns of selected molecles based on transcriptome analysis.** P0-Cre/Floxed-EGFP mouse embryos at E10.5 were immunostained (A–C). In the periocular mesenchyme as a representative craniofacial region, EGFP^+^ cells were positive for PDGFRα and PDGFRβ but negative for Sox10 (D–F). In the dorsal root ganglion (DRG) as a representative trunk region, EGFP^+^ cells were positive for Sox10, but negative for PDGFRα and PDGFRβ.
(TIF)

Figure S3 **Immunohistochemistry of NCC markers.** P0-Cre/Floxed-EGFP mouse embryos at E10.5 were immunostained (A, C, E). In the periocular mesenchyme as a representative craniofacial region, EGFP cells were positive for p75 but negative for NCAM and N-Cad (B, D, F). In the neural tissues as a representative trunk region, EGFP cells were positive for NCAM, N-Cad and p75.
(TIF)

Table S1 **The 347 differentially expressed genes for clustering analyses.** The following data are shown for each probe; probe ID, gene symbol of the corresponding gene, Log2 expression values in the eight samples including replicates, p value from ANOVA, Benjamini-Hochberg FDR, average expression values groups in the four tissue, expression range among the four tissue groups, and the cluster ID in Figure 3.
(XLSX)

Table S2 **Top 10 enriched Gene Ontology Biological Process terms for cluster A.**
(DOCX)

Table S3 **Top 10 enriched Gene Ontology Biological Process terms for cluster C.**
(DOCX)

Table S4 **Top 10 enriched Gene Ontology Biological Process terms for cluster D.**
(DOCX)

Table S5 **Top 10 enriched Gene Ontology Biological Process terms for cluster E.**
(DOCX)

Table S6 **Top 10 enriched Gene Ontology Biological Process terms for cluster F.**
(DOCX)

Table S7 **Top 10 enriched Gene Ontology Biological Process terms for cluster G.**
(DOCX)
