# Supplementary figures and images for: Molecular and Cellular Features of Murine Craniofacial and Trunk Neural Crest Cells as Stem Cell-Like Cells
Source: PLoS One. 2014 Jan 20;9(1):e84072. doi: 10.1371/journal.pone.0084072 (PMC3896334; doi:10.1371/journal.pone.0084072)

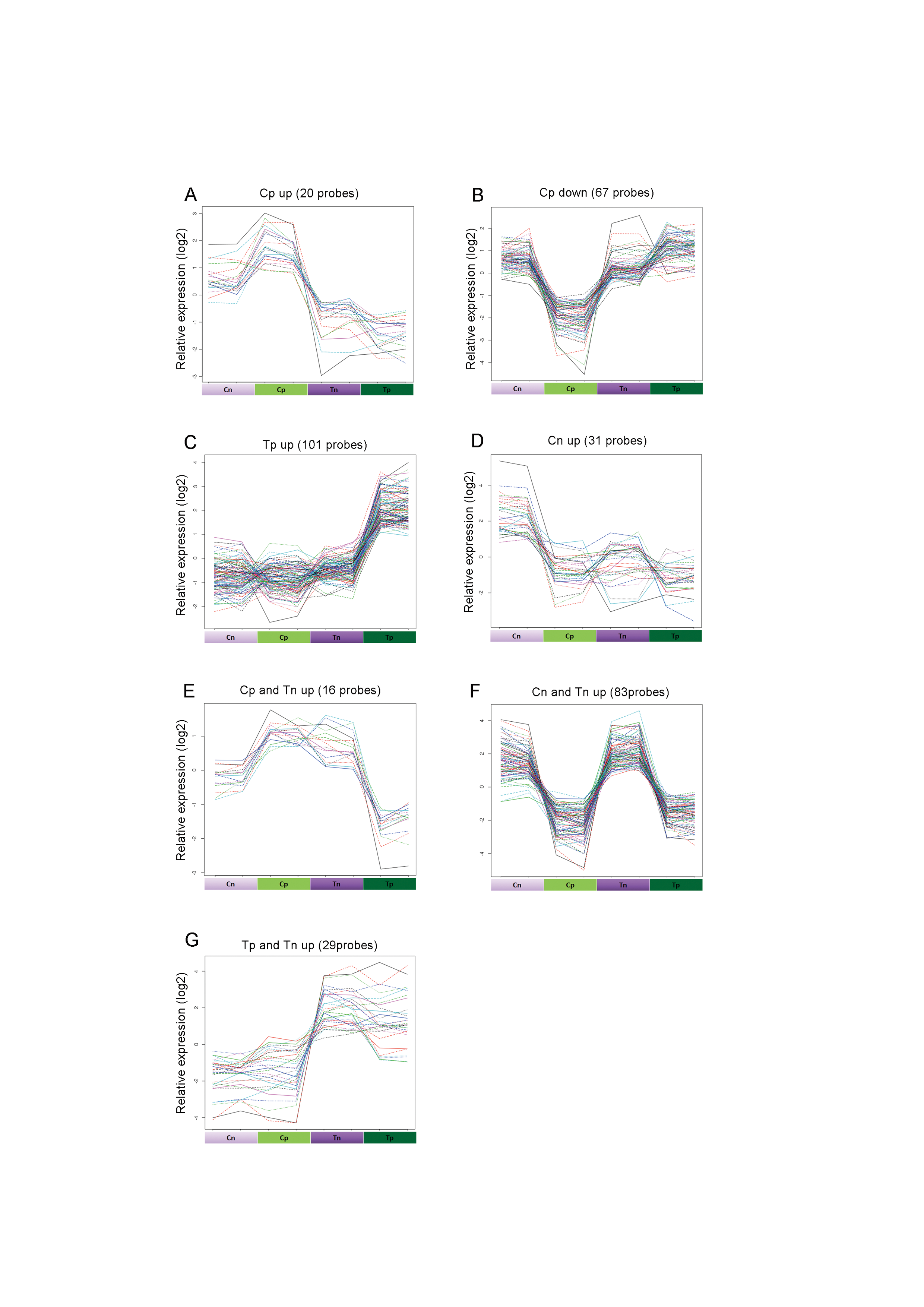

Supplement: Figure S1 — Clustering on the data sets from general filtering of four populations. From these four populations (Cp, Tp, Cn, Tn), we identified 7 probe clusters. (A) Cp up-regulation (B) Cp down-regulation (C) Tp up-regulation (D) Cn up-regulation (E) Cp and Tp up-regulation (F) Cn and Tn up-regulation (G) Tp and Tn up-regulation. (TIF) [file pone.0084072.s001.tif]

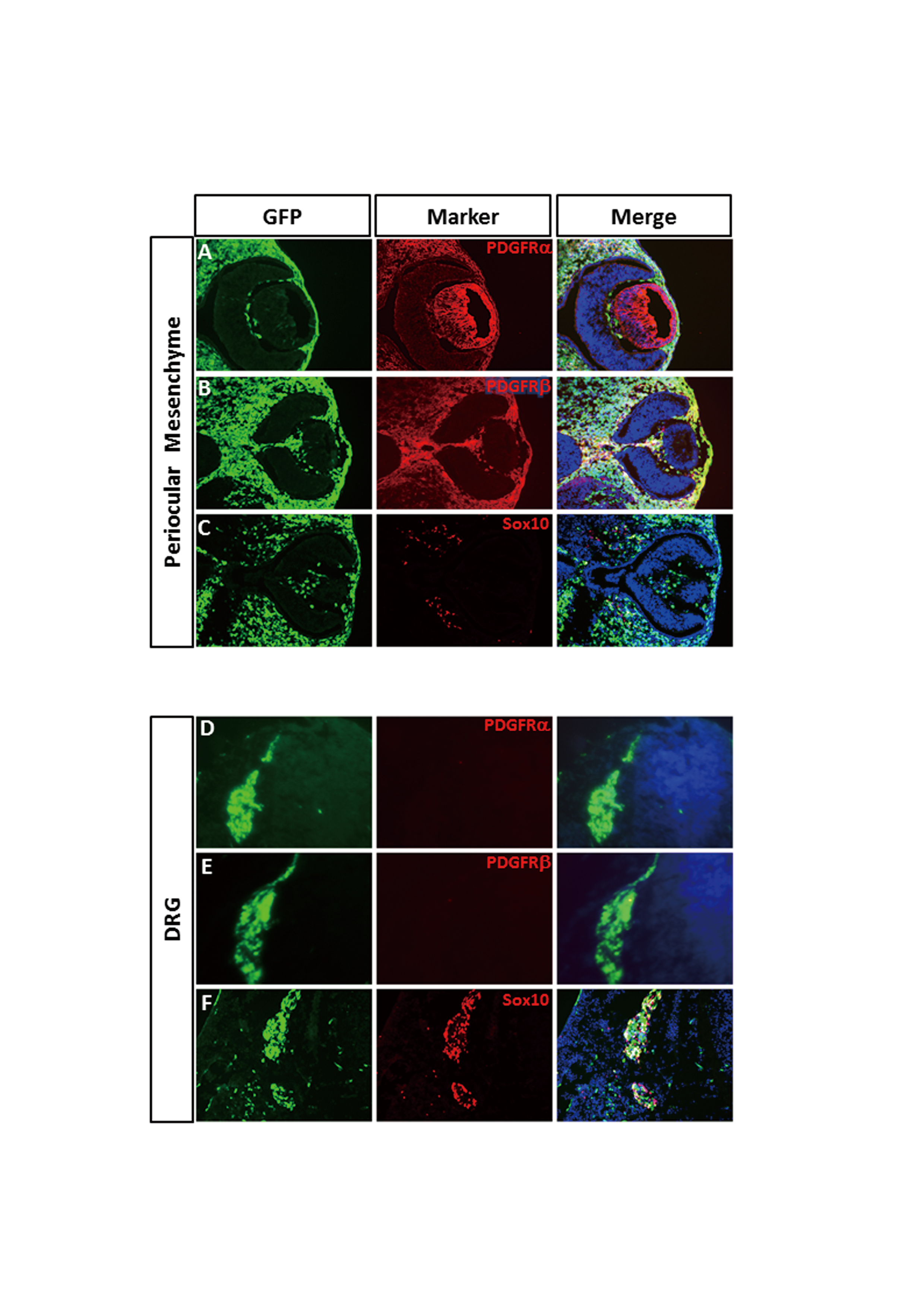

Supplement: Figure S2 — Expression patterns of selected molecles based on transcriptome analysis. P0-Cre/Floxed-EGFP mouse embryos at E10.5 were immunostained (A–C). In the periocular mesenchyme as a representative craniofacial region, EGFP+ cells were positive for PDGFRα and PDGFRβ but negative for Sox10 (D–F). In the dorsal root ganglion (DRG) as a representative trunk region, EGFP+ cells were positive for Sox10, but negative for PDGFRα and PDGFRβ. (TIF) [file pone.0084072.s002.tif]

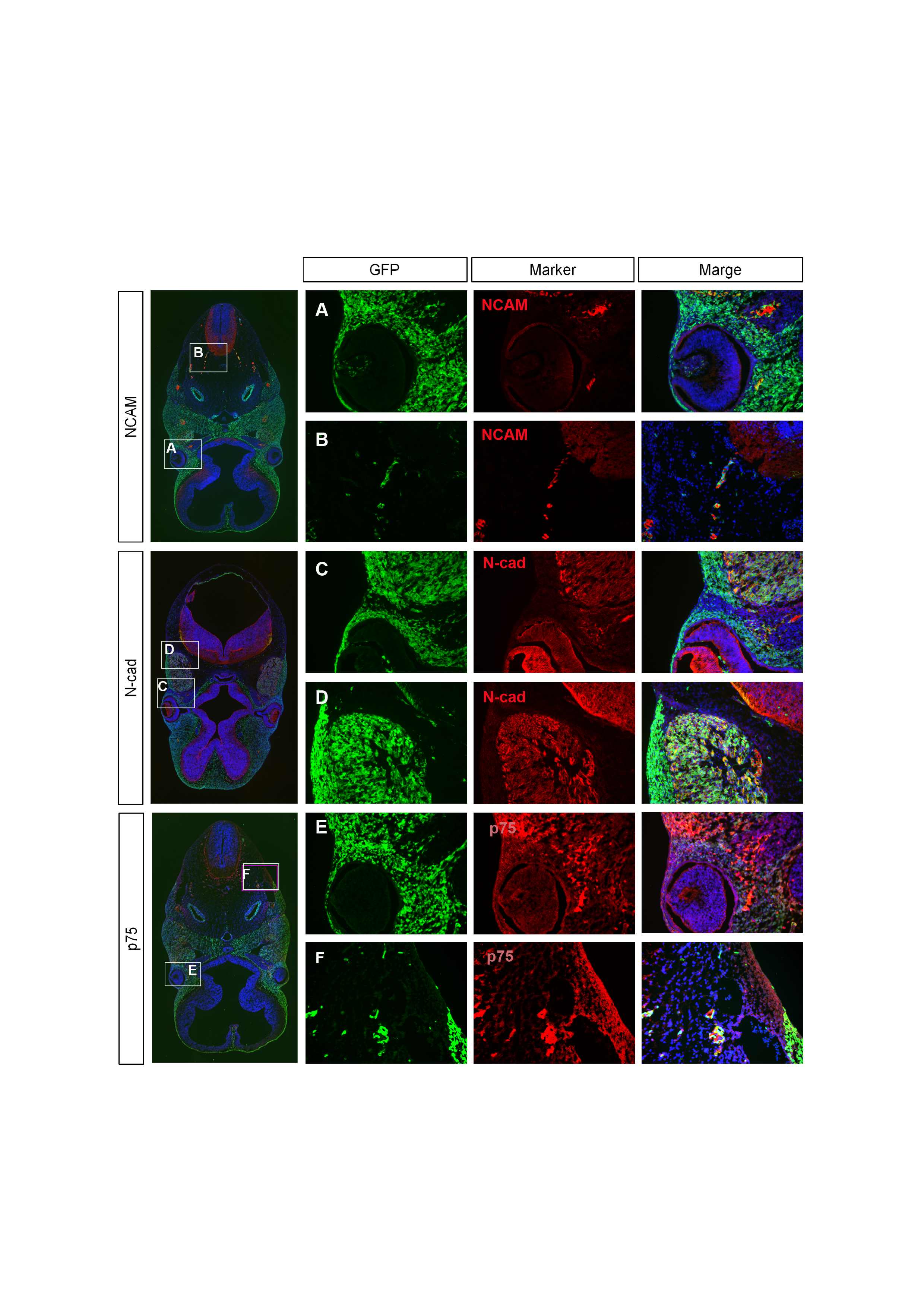

Supplement: Figure S3 — Immunohistochemistry of NCC markers. P0-Cre/Floxed-EGFP mouse embryos at E10.5 were immunostained (A, C, E). In the periocular mesenchyme as a representative craniofacial region, EGFP cells were positive for p75 but negative for NCAM and N-Cad (B, D, F). In the neural tissues as a representative trunk region, EGFP cells were positive for NCAM, N-Cad and p75. (TIF) [file pone.0084072.s003.tif]
