# Supplementary material for: Molecular and Cellular Features of Murine Craniofacial and Trunk Neural Crest Cells as Stem Cell-Like Cells
Source: PLoS One. 2014 Jan 20;9(1):e84072. doi: 10.1371/journal.pone.0084072 (PMC3896334; doi:10.1371/journal.pone.0084072)
Supplement: Table S2 — Top 10 enriched Gene Ontology Biological Process terms for cluster A. (DOCX) [file pone.0084072.s005.docx]

**Table S2** Top 10 enriched Gene Ontology Biological Process terms for cluster A

| GO ID | Category | ­Number of genes | p value |
| --- | --- | --- | --- |
| 9888 | tissue development | 6 | 3.6E-04 |
| 30154 | cell differentiation | 8 | 3.6E-04 |
| 6355 | regulation of transcription, DNA-dependent | 7 | 3.6E-04 |
| 30111 | regulation of Wnt receptor signaling pathway | 3 | 4.1E-04 |
| 30326 | embryonic limb morphogenesis | 3 | 1.1E-03 |
| 60429 | epithelium development | 4 | 1.2E-03 |
| 30855 | epithelial cell differentiation | 3 | 1.4E-03 |
| 60828 | regulation of canonical Wnt receptor signaling pathway | 2 | 1.7E-03 |
| 30178 | negative regulation of Wnt receptor signaling pathway | 2 | 3.0E-03 |
| 3308 | negative regulation of Wnt receptor signaling pathway involved in heart development | 1 | 5.6E-03 |
