# Supplementary material for: Molecular and Cellular Features of Murine Craniofacial and Trunk Neural Crest Cells as Stem Cell-Like Cells
Source: PLoS One. 2014 Jan 20;9(1):e84072. doi: 10.1371/journal.pone.0084072 (PMC3896334; doi:10.1371/journal.pone.0084072)
Supplement: Table S3 — Top 10 enriched Gene Ontology Biological Process terms for cluster C. (DOCX) [file pone.0084072.s006.docx]

**Table S3** Top 10 enriched Gene Ontology Biological Process terms for cluster C

| GO ID | Category | ­Number of genes | p value |
| --- | --- | --- | --- |
| 7399 | nervous system development | 25 | 4.0E-14 |
| 22008 | neurogenesis | 18 | 9.0E-11 |
| 30182 | neuron differentiation | 14 | 5.3E-09 |
| 7409 | axonogenesis | 8 | 4.0E-06 |
| 10001 | glial cell differentiation | 5 | 2.8E-05 |
| 7610 | behavior | 10 | 4.0E-05 |
| 40011 | locomotion | 9 | 4.5E-05 |
| 6928 | cellular component movement | 9 | 6.4E-05 |
| 45664 | regulation of neuron differentiation | 7 | 6.6E-05 |
| 48513 | organ development | 17 | 1.6E-04 |
