# Supplementary material for: Molecular and Cellular Features of Murine Craniofacial and Trunk Neural Crest Cells as Stem Cell-Like Cells
Source: PLoS One. 2014 Jan 20;9(1):e84072. doi: 10.1371/journal.pone.0084072 (PMC3896334; doi:10.1371/journal.pone.0084072)
Supplement: Table S4 — Top 10 enriched Gene Ontology Biological Process terms for cluster D. (DOCX) [file pone.0084072.s007.docx]

**Table S4** Top 10 enriched Gene Ontology Biological Process terms for cluster D

| GO ID | Category | ­Number of genes | p value |
| --- | --- | --- | --- |
| 32502 | developmental process | 22 | 3.59E-16 |
| 30900 | forebrain development | 11 | 8.81E-16 |
| 30182 | neuron differentiation | 11 | 4.70E-12 |
| 19222 | regulation of metabolic process | 17 | 4.98E-10 |
| 10468 | regulation of gene expression | 15 | 1.68E-09 |
| 9653 | anatomical structure morphogenesis | 12 | 2.84E-09 |
| 7389 | pattern specification process | 8 | 2.87E-09 |
| 3002 | regionalization | 7 | 1.97E-08 |
| 9887 | organ morphogenesis | 9 | 4.27E-08 |
| 48598 | embryonic morphogenesis | 7 | 5.02E-07 |
