# Supplementary material for: Molecular and Cellular Features of Murine Craniofacial and Trunk Neural Crest Cells as Stem Cell-Like Cells
Source: PLoS One. 2014 Jan 20;9(1):e84072. doi: 10.1371/journal.pone.0084072 (PMC3896334; doi:10.1371/journal.pone.0084072)
Supplement: Table S6 — Top 10 enriched Gene Ontology Biological Process terms for cluster F. (DOCX) [file pone.0084072.s009.docx]

**Table S6** Top 10 enriched Gene Ontology Biological Process terms for cluster F

| GO ID | Category | ­Number of genes | p value |
| --- | --- | --- | --- |
| 14706 | striated muscle tissue development | 9 | 1.07E-08 |
| 7519 | skeletal muscle tissue development | 7 | 3.23E-08 |
| 2376 | immune system process | 14 | 3.23E-08 |
| 51146 | striated muscle cell differentiation | 7 | 1.08E-07 |
| 55002 | striated muscle cell development | 6 | 2.23E-07 |
| 6935 | chemotaxis | 7 | 2.33E-07 |
| 15669 | gas transport | 4 | 3.06E-07 |
| 6936 | muscle contraction | 6 | 3.09E-07 |
| 30593 | neutrophil chemotaxis | 4 | 3.20E-06 |
| 6941 | striated muscle contraction | 4 | 1.11E-05 |
