# Supplementary material for: Molecular and Cellular Features of Murine Craniofacial and Trunk Neural Crest Cells as Stem Cell-Like Cells
Source: PLoS One. 2014 Jan 20;9(1):e84072. doi: 10.1371/journal.pone.0084072 (PMC3896334; doi:10.1371/journal.pone.0084072)
Supplement: Table S7 — Top 10 enriched Gene Ontology Biological Process terms for cluster G. (DOCX) [file pone.0084072.s010.docx]

**Table S7** Top 10 enriched Gene Ontology Biological Process terms for cluster G

| GO ID | Category | ­Number of genes | p value |
| --- | --- | --- | --- |
| 9952 | anterior/posterior pattern formation | 15 | 1.64E-26 |
| 48706 | embryonic skeletal system development | 13 | 1.55E-25 |
| 48704 | embryonic skeletal system morphogenesis | 12 | 1.24E-24 |
| 6355 | regulation of transcription, DNA-dependent | 15 | 1.20E-14 |
| 34645 | cellular macromolecule biosynthetic process | 15 | 3.31E-11 |
| 51216 | cartilage development | 3 | 3.28E-04 |
| 30326 | embryonic limb morphogenesis | 3 | 4.34E-04 |
| 30878 | thyroid gland development | 2 | 5.32E-04 |
| 9954 | proximal/distal pattern formation | 2 | 1.22E-03 |
| 35136 | forelimb morphogenesis | 2 | 1.22E-03 |
